# How the humanities shape medical culture: Knowing Wegener and other Nazi eponyms

**DOI:** 10.15694/mep.2018.0000175.1

**Published:** 2018-08-21

**Authors:** Lester Liao, Dax Gerard Rumsey

**Affiliations:** 1 University of Alberta

**Keywords:** Medical Humanities, Nazism, Nazi Doctors, Wegener, Reiter, Ethics, History, Curricular reform, Eponyms, Medical Education, Reductionism, Hidden Curriculum, Medical Culture, Conscience, Humanism

## Abstract

This article was migrated. The article was marked as recommended.

While the medical humanities have experienced a renaissance, they are still largely a peripheral component of medical education. This is troublesome because the humanities include a number of disciplines that are foundational in understanding medicine and how it should be practiced. Nonetheless, current medical culture makes it difficult to fully incorporate the humanities into curriculum. We therefore propose an incremental approach to shaping the medical culture that can easily be incorporated into daily teaching as opposed to designing additional classes and resources that must be added to existing educational structures. An example of this approach is reviewed here through teaching historical and ethical lessons surrounding Nazi eponyms. The use of names like Wegener provide brief opportunities for sidebars during clinical lectures to remind learners that empirical data do not provide ethical direction and that our medical history has included atrocities that remind us to practice conscientiously. We provide other examples that can be included in daily learning. This approach eschews the burdens associated with large curricular changes, such as student resistance/apathy and logistical barriers, and can be easily implemented. It also enables change to be gradual and through structures that have already been established, allowing learners to see the benefits of insights from the humanities in small, digestible segments. Through this approach, medical culture can be shaped towards a greater appreciation toward the medical humanities.

## Introduction

Medical learners today are increasingly out of touch with the humanities.  When they arrive at medical school, few have spent any significant time reading about the history of humanity or philosophy.  They have instead been steeped in specialized and technical knowledge related to how the human body works or various other scientific disciplines.  Universities once educated their students about the universe, but now they have ruptured into studies of separate spheres of knowledge. Students are unable to see how various disciplines find “their true place in the universal system… [and] their mutual dependence,” leaving a fragmentary vision of the world ( Newman, 1996).  Hence they arrive with skepticism toward the value of history and philosophy.  They often fail to see that their very practice of medicine depends on values and a cultural epoch that cannot be isolated from the rest of humanity’s history ( Ferry, 2011).  This leads to reductionistic medicine.

Much can be said about how this trend can be challenged.  Our current trajectory is a reflection of far more than the culture of medicine.  It is a progression of the autocratic and pragmatic philosophy that has ensnared Western society ( Marsden, 1996).  Yet the situation is not without hope. Medical humanities have experienced a renaissance ( Bleakley, 2015; Cole, Carlin and Carson, 2015), and it is only through their incorporation into the daily culture and teaching of medicine that their benefits can be reaped.  Simply adding a separate class on the humanities will not do.  In the age of checking off boxes and buffing up resumes, students will do what needs to be done, but this will hardly lead to personal change ( Deresiewicz, 2014).  What we need is a cultural shift, a change in the subtle things about how we approach medicine in our perfunctory tasks.

## Historical Eponyms and their Lessons

One of the simplest ways to cultivate an appreciation for medical humanities and its impact on contemporary medical practice is through historical eponyms for diseases.  While it is true that some names are in need of revision due to previous errors (e.g. a disease named after someone other than the original describer), it is still useful to contemplate how various labels originated and the context that encouraged their development.  Various eponyms provide potential for brief historical and moral lessons without being an onerous addition to the curriculum.  Particularly for audiences or teachers whose tolerance for the humanities is low, these can become memorable five-minute sidebars in clinical lectures.

Most notably, the term Wegener’s granulomatosis is currently being phased out of use and replaced by descriptive nomenclature ( Falk *et al.*, 2011). This recommendation was in part triggered by concerns that Wegener was involved with the Nazi Party ( Woywodt *et al.*, 2006).  Others have also advocated for replacing the term Reiter syndrome with reactive arthritis ( Keynan and Rimar, 2008).  Despite these changes, we believe that these names are of crucial importance for learners as historical lessons even if they are no longer used in the clinical setting. This is because the simple utterance of “Wegener” brings to mind a Nazi.  There were physicians – doctors like you and me – that were Nazis.  This must never be forgotten.

Wegener reminds us that the history of medicine is not pristine.  Medicine has a tremendous capacity to do good, but it likewise can be a tool for evil.  This capacity lies not in medicine itself but in the people who practice medicine.  The Nazi doctors were at the cutting edge of the medical world, and yet their actions were amongst the most deplorable in history ( Lifton, 1986; Spitz, 2005).  As we obsess over the newest technological advancements or medical capabilities, we must not forget that new moral questions arise.  Emphasis on progress must be tempered by an accurate sense of what progress actually entails.  It is not progress to gain an understanding of disease pathology through spilled blood and disregard for human dignity: this is an egregious regression.  When on the trail of error, progress necessitates turning around and going in the opposite direction.  It requires withholding the knife and aborting the project.  We should celebrate medical innovation, but we must not forget that empirical data give no guidance on how to practice ethically ( Liao, 2017).  Drugs cannot administer themselves; people administer drugs.

Two more simple lessons can be gleaned from a term like Wegener’s.  First, there was, as the German-born Jewish philosopher Hannah Arendt said, a certain “banality to evil” ( Arendt, 1965).  As Eichmann famously declared at the Nuremberg trials, he was simply following orders.  Perhaps more disturbingly, he simply followed the law.  This should make contemporary physicians at least think twice about simply doing what is legally mandated or culturally popular.

We must be wary of two mechanisms.  The first occurs with a single clinical decision when we abdicate our moral sensibilities for the sake of expediency or external pressure.  This injury to conscience is grave, but repeated offenses of this nature have a numbing effect until we no longer object and simply behave as a robot would.  Courage is critical in mitigating this chain of events.  The second mechanism is more sinister.  It includes clever, manipulative rhetoric that frames an evil as a good such that it becomes desirable.  In Germany this included propaganda of a utopian vision alongside the “problem” of those who were portrayed as subhuman and the necessity to eliminate them ( Herf, 2008).  This rhetoric often begins subtly and grows bolder as fence-sitters become increasingly captivated by a particular vision.  Physicians can resist such attempts through an awareness of history and how these regimes develop.

Another lesson involves understanding the characteristics of Nazi physicians like Wegener.  While the extremity of Nazi murder “renders it close to unreality,” it was unquestionably real ( Lifton, 1986).  In describing the Nazi doctors, the psychiatrist Dr. Robert Jay Lifton in his landmark book, *The Nazi Doctors,* said, “They were by no means the demonic figures – sadistic, fanatic, lusting to kill – people have often thought them to be… ordinary people can commit demonic acts” ( Lifton, 1986). Arendt had similarly noted how normal Eichmann was; he had no mental illness.  Normal people can do bad things.  Contemporary physicians must not naively believe they are incapable of evil.  It is those who believe themselves least capable of committing atrocities who are the least cautious and most worrisome.

Trainees must not forget that our heritage has involved serious evil.  This is not esoteric history but relevant to all entering the profession.  These terms do not confer honour, as they are names to which we should react to as we would to Mengele and other Nazi names.  We remember instead tragedy.  Learning these names will teach us much, like the history of mandatory sterilizations or Tuskegee.  We must be unassuming of our own morality and ever vigilant over our practices.

To be clear, we are not advocating to discard granulomatosis with polyangiitis or reactive arthritis.  These are clinically important terms that we wholeheartedly endorse.  Yet the move toward strictly pathophysiological language must not reflect an orientation that believes medicine is somehow just about science and empirical data and that all sociohistorical phenomena can be discarded.  Medicine is about people.  And so we simply encourage that, in the educational context, learners be reminded of historical names and their origins.  For in remembering our (sometimes blemished) past, we foster a collective conscience in medicine for ethical practice in the future.

## Conclusion

It will certainly prove to be a challenge to incorporate the humanities more broadly into medicine, but here we have provided one small example of how we can instill important insights into medical education through eponyms.  Further opportunities exist through reviewing historical discoveries (e.g. the role of luck in penicillin), key figures in medical history (e.g. Galen’s desertion during epidemics), evolving treatment regimens (e.g. cardiac repair and societal perceptions of Down syndrome), and many other avenues.  While ideally larger segments of time and energy could be dedicated to exploring the humanities in medicine, we suspect the majority of students would be less than enthusiastic for such learning.  Perhaps with small segments of history and philosophy embedded into daily learning, students can begin to see how these disciplines are not only relevant but foundational to medical practice.

## Take Home Messages

• Medical learners are in need of greater exposure to the humanities.
• Due to systemic barriers surrounding curricular reform and the general skepticism of many learners towards the humanities, a new approach is needed to encourage their role in medical education.
• The medical humanities can be effectively taught through embedding historical and ethical insights into daily learning, contributing to a change in medical culture.
• Nazi eponyms provide an opportunity to discuss historical lessons within daily clinical lectures.
• Historical and ethical insights from the humanities can help learners to practice conscientiously for the future.

## Notes On Contributors

Dr Lester Liao is a pediatric resident at the University of Alberta. He currently acts as the Resident Lead for the Arts and Humanities in Health and Medicine program and as the resident affiliate for the John Dossetor Health Ethics Centre.Dr. Dax Rumsey is an assistant professor in the Department of Pediatrics at the University of Alberta. He is a staff pediatric rheumatologist at the Stollery Children’s Hospital.
