# Supplementary material for: How the humanities shape medical culture: Knowing Wegener and other Nazi eponyms
Source: MedEdPublish (2016). 2018 Aug 21;7:175. [Version 1] doi: 10.15694/mep.2018.0000175.1 (PMC10701796; doi:10.15694/mep.2018.0000175.1)
Supplement: Peer review history

# Reviewer report: Reviewer response for version 1

**DOI:** 10.21956/mep.19645.r29286

**Published:** 2018-09-04

**Authors:** P Ravi Shankar

**Affiliations:** 1 American International Medical University

This review has been migrated. The reviewer awarded 4 stars out of 5 This is an interesting and well-written article. I do agree with the authors that knowledge of the history of medicine and of the humanities among modern medical students is low. Among the students whom I teach only a few are aware of the important historical figures in medicine. These are rarely taught and are regarded as unimportant by students and teachers. These are not assessed during licensing exams and do not receive much attention. In South Asia admission to medical school is restricted to students from a science background. The authors describe how eponyms can be used to introduce students to the history of medicine and other issues. The case of Wegener has been well described. Evil deeds can often be carried out by ‘good’ people who were just carrying out orders. The ability for ethical reasoning should be developed among medical students. The approach described by the authors can strengthen the learning of the medical humanities and intertwine it with the history of medicine.

Reviewer Expertise:

NA

No decision status is available

# Reviewer report: Reviewer response for version 1

**DOI:** 10.21956/mep.19645.r29287

**Published:** 2018-09-04

**Authors:** Gert Van Zyl

**Affiliations:** 1 University of the Free State

This review has been migrated. The reviewer awarded 5 stars out of 5 A very sensitive area of history turned into a very important learning activity. I really enjoyed reading this piece and found it to be of value to all environments of medical education. It makes a valid point about the lack of knowledge on humanities and history of students and the role of humanities in education with good examples and practical application. Well done!

Reviewer Expertise:

NA

No decision status is available

# Reviewer report: Reviewer response for version 1

**DOI:** 10.21956/mep.19645.r29285

**Published:** 2018-08-29

**Authors:** Hedy S. Wald

**Affiliations:** 1 Warren Alpert Medical School of Brown University

This review has been migrated. The reviewer awarded 4 stars out of 5 “There were physicians – doctors like you and me – that were Nazis. This must never be forgotten.” The essence of this important paper. This resonated for me with our paper published in Hektoen (Wald et al, 2017) on - “Holocaust as End Stage Disease – Medical Education as a Moral Imperative” http://hekint.org/2017/01/29/the-holocaust-as-end-stage-disease-medical-education-as-a-moral-imperative/ underlining the point of “doctors like you and me” and highlighting the relevance of this history for the here and now of clinical and research medicine. Thank you Drs. Liao and Rumsey for highlighting this crucial history of medicine (as part of the medical humanities) and advocating for its inclusion in medical education to help foster ethical vigilance and moral resilience. Helping our learners to confront how German physicians enrolled in the Nazi party at a rate three times higher than any other profession (Caplan, 1989, Hastings Cent Rep) and the sheer magnitude of involvement in atrocities (including Aktion T4 euthanasia program to exterminate the disabled and medicalized genocide). Yet another powerful example of the way medical humanities can play such an integral role in medical education. Guided reflection on this history and its implications for one’s own professionalism and professional identity formation can ideally help equip our learners (and ourselves) when facing and grappling with contemporary ethical dilemmas in medicine. I might suggest some additional papers within the literature review (references) which relate to this topic area within medical education, including “Learning from the past” – medicine and the Holocaust (2009, Lancet, Reis & Wald) https://www.thelancet.com/journals/lancet/article/PIIS0140-6736(09)61275-7/abstract and Bruns & Chelouche (2017, Annals Int Med) “Lectures on Inhumanity: Teaching Medical Ethics in German Medical Schools Under Nazism” which highlighted that “medical ethics in Nazi-era medical school curricula existed yet included “unequal worth of human beings, authoritative role of the physician, and priority of public health over individual -patient care” with the latter resonating hauntingly with current concerns about cultivating and maintaining person-centered care. Eponyms of Nazi-era physicians sadly exist with various specialties and may be powerful exemplars for curriculum inclusion within undergraduate and/or graduate medical education (examples of references include Kondziella & Zeidman 2016, Front Neurol Neurosci, “What’s in a Name? Neurological Eponyms of the Nazi Era”; Eckert et al, 2018 J Am Coll Radiology “Facing the Guilt and Commemorating the Victims: German Radiology and Radiation Oncology During National Socialism, and Halioua, 2010, J Low Genit Tract Dis, “The participation of Hans Hinselmann in medical experiments at Auschwitz”). A community of healthcare professionals, health professions educators, humanities scholars and historians have signed the Galilee Declaration calling for this education in all healthcare professions schools (http://english.wgalil.ac.il/category/Declaration)In addition to inclusion of additional relevant references, this paper could be strengthened with inclusion of ideas on curriculum implementation at both UME and GME levels, which would ideally include facilitated reflection. An example of curriculum innovation is provided in Reis & Wald, 2015, “Contemplating medicine during the Third Reich: scaffolding professional identity formation for medical students” and others may be found on http://www.medicineaftertheholocaust.org/ Thank you for this valuable contribution on such an important topic in ethics, professionalism and professional identity formation in the “becoming” of a physician.

Reviewer Expertise:

NA

No decision status is available

# Reviewer report: Reviewer response for version 1

**DOI:** 10.21956/mep.19645.r29282

**Published:** 2018-08-28

**Authors:** Gary D. Rogers

**Affiliations:** 1 Griffith University

This review has been migrated. The reviewer awarded 5 stars out of 5 This well-written opinion piece reports an example of the rare jewel that is a genuinely ‘really good idea’ for health professional education practice. The authors suggest a truly clever way to engage students with historical stories that raise important ethical issues as a part of routine biomedical learning. Appropriately, they propose that these elements be included for all students. This approach offers a much preferable alternative to the highly elaborate medical humanities programs that some institutions have devised but offer only on a voluntary basis, thus ensuring their delivery only to the students who actually need them least!The authors have used the eponymous connections between some medical syndromes and doctors known to have Nazi sympathies as an example but point out that there are many similar opportunities to connect original descriptions of pathological conditions (or ‘research’ activities or treatment discoveries) to their historical contexts and attendant ethical considerations.I would be particularly interested to hear how the authors plan to assess the knowledges and ethical understandings derived from these ‘side-bars’, in order to encourage student engagement further.I would encourage the authors to work up this concept into a detailed curriculum, implement it and then evaluate its impact and report their findings in the literature. Qualitative approaches, such as phenomenologically-derived techniques that focus on students’ experiences of the approach and the affective learning they generate, would be particularly appropriate to this evaluation task. I will look forward to reading more about this great educational idea.

Reviewer Expertise:

NA

No decision status is available

# Reviewer report: Reviewer response for version 1

**DOI:** 10.21956/mep.19645.r29284

**Published:** 2018-08-23

**Authors:** Jonathan McFarland

**Affiliations:** 1 Sechenov University, Moscow

This review has been migrated. The reviewer awarded 3 stars out of 5 An interesting article on a very delicate subject. I firmly agree with the main thesis of the paper, and that is that we can learn from the past, both the good and the bad. In actual fact, this is a priority, we need to learn from the mistakes of the past, and perhaps this is even more important in medicine. And, I agree wholeheartedly with the authors when they say that medicine is about people, and that by remembering our sometimes "blemished" past we can foster a collective conscience for future ethical practice. We must remember, we must never forget. However, I would be wrong if I did not say I have some doubts because the line between learning from and honouring does not seem straightforward, and, in line with the other reviewers, I think that this could be further developed, and when it is, then I would be extremely interested to see it. It has certainly given us some food for thought and should, I hope, raise a lot of interest and debate. Thank you

Reviewer Expertise:

NA

No decision status is available

# Reviewer report: Reviewer response for version 1

**DOI:** 10.21956/mep.19645.r29283

**Published:** 2018-08-21

**Authors:** Ken Masters

**Affiliations:** 1 Sultan Qaboos University

This review has been migrated. The reviewer awarded 3 stars out of 5 A delicate subject, well-handled, (and I say this also as person currently living in Austria, just a stone’s throw from Mauthausen), although I felt that the authors should have taken it further in order to make a convincing argument and greater value to the reader. Sometimes, the argument appears to meander a little, although the general gist of directly addressing issues of Nazi medicine (if one can call it that), in order to learn about medical practice, and its practitioners, in general, is made. The authors have stopped rather short of where I felt they could have taken this; perhaps they were reticent to go into detail because of the delicate nature of the topic, but I would like to have seen have seen further expansion:• The authors touch on well-known examples, which is good to introduce the topic, but could have raised others that are less well-known, and also touched on related sociological studies (as the authors are advocating for more humanities in medical education), in Germany at the time, such as the work by Eva Justin on “Gypsy” children).• Broadening the context a little to similar, but less known (and far less acknowledged) medical experiments performed in Japan over the same period. I realise that the authors are confining themselves to the Nazi practices, but, by drawing in these examples, the authors can look for comparisons and principles, and demonstrate that this is not merely a “German” thing, but goes beyond national boundaries. (Their reference to Tuskegee does that to some extent, but it really should go further). The Japanese example is particularly interesting, because the doctors attempted to use the results of their experiments as bargaining chips to receive lighter sentences. One can imagine the ethical complexities involved in that.• Understanding the complexity of historical, legal and social context, and reflecting on what future generations of medical students might think of our practices that we currently hold as acceptable. It would be useful exercise for medical students to look at current gold practices through the lenses of a future generation.• Examples: It would have been useful if the authors could describe one or two practical examples of how this historical context could be (or has been) used in teaching medical students.

Reviewer Expertise:

NA

No decision status is available

# Reviewer report: Reviewer response for version 1

**DOI:** 10.21956/mep.19645.r29281

**Published:** 2018-08-21

**Authors:** Trevor Gibbs

**Affiliations:** 1 AMEE

This review has been migrated. The reviewer awarded 3 stars out of 5 As someone who qualified 45 years ago, I can already reflect on treatments widely accepted when I was a student, that are seriously looked down upon now ( aversion therapy and for homosexuality, nil therapy for Downs syndrome babies to name but two), so I would definately agree with looking back at practices and revisiting their ethics. I would certainly agree with looking back over history and observing the rights and wrongs of other famous and infamous doctors- it is important for students to think and reflect upon these. I am a little uncomfortable with just looking at the notorious German doctors given that there are many more scattered around the world. So although I think that this paper has merit in making us look back to look forward , I would prefer that it had a wider scope.I was waiting for a more practical ending to this paper. I can feel the passion expressed by the author but we also need not just contemplative reflection but how one puts these aspects of humanities teaching into practice.

Reviewer Expertise:

NA

No decision status is available
